# Anisotropic source modelling for turbulent jet noise prediction

**DOI:** 10.1098/rsta.2019.0075

**Published:** 2019-10-14

**Authors:** Xihai Xu, Xiaodong Li

**Affiliations:** 1 School of Aeronautic Science and Engineering, Beihang University (BUAA), Beijing 100191, People's Republic of China; 2 School of Energy and Power Engineering, Beihang University (BUAA), Beijing 100191, People's Republic of China

**Keywords:** jet noise, acoustic analogy, turbulent noise, turbulence model

## Abstract

An anisotropic component of the jet noise source model for the Reynolds-averaged Navier–Stokes equation-based jet noise prediction method is proposed. The modelling is based on Goldstein's generalized acoustic analogy, and both the fine-scale and large-scale turbulent noise sources are considered. To model the anisotropic characteristics of jet noise source, the Reynolds stress tensor is used in place of the turbulent kinetic energy. The Launder–Reece–Rodi model (LRR), combined with Menter's *ω*-equation for the length scale, with modified coefficients developed by the present authors, is used to calculate the mean flow velocities and Reynolds stresses accurately. Comparison between predicted results and acoustic data has been carried out to verify the accuracy of the new anisotropic source model.

This article is part of the theme issue ‘Frontiers of aeroacoustics research: theory, computation and experiment’.

## 1. Introduction

Since the 1950s, jet noise has been a classic research issue in the field of aeroacoustics. Many researchers have devoted themselves to the study of jet noise generation and radiation mechanisms, noise prediction methods and low noise design. The study of jet noise directly contributed to the emergence and development of aeroacoustics. In 1952 and 1954, Lighthill [1,2] provided a systematic basis for predicting jet noise when he rearranged the Navier–Stokes equations into the form of a linear wave equation for a medium at rest with a quadrupole-type source term. The crucial step in this so-called acoustic analogy approach amounts to assuming that the source term is in some sense known or that it can at least be modelled in an approximate fashion. Early efforts to improve the Lighthill approach focused on accounting for mean flow interaction effects. Pridmore-Brown [3], Phillips [4], Lilley [5] and many others sought to accomplish this by rearranging the Navier–Stokes equations into the form of an inhomogeneous convective, or moving medium, wave equation rather than the inhomogeneous stationary medium wave equation originally proposed by Lighthill. Jet noise theory is primarily concerned with predicting acoustic spectra. The methods for predicting the noise radiated by a turbulent flow can be divided into two categories: the direct prediction methods and the hybrid prediction methods. The direct prediction methods, such as direct numerical simulation (DNS), large eddy simulation (LES) and detached eddy simulation (DES), were developed rapidly in recent years. However, these simulations are computationally expensive and time-consuming. Therefore, the hybrid prediction methods based on the solutions of steady Reynolds-averaged Navier–Stokes (RANS) equations and half empirical acoustic models are still the dominant methods for jet noise prediction. For subsonic jets, large-scale turbulent structures and fine-scale turbulence are thought to be the main noise sources. Since Lighthill's pioneering work on aerodynamic noise, most of the jet noise prediction methods have been based on the acoustic analogy. The hybrid jet noise prediction methods, based on mean flow and turbulence information and the acoustic models, have been studied extensively, most of which are on the basis of acoustic analogy. In 1978, based on Lilley's [3] equation, Balsa *et al*. [6] developed a RANS-based Mani–Glieb–Balsa approach (MGB) to predict the jet noise, which became a popular industrial noise prediction tool. In the 1990s, by improving the jet noise source model, Khavaran advanced an MGB approach to the Mani–Glieb–Balsa–Khavaran (MGBK) method [7,8]. In 1999, Tam & Auriault [9] developed a theory of predicting fine-scale turbulence jet noise from cold to moderate temperature jet. In 2005, a more general source model was introduced by Tam & Pastouchenko [10] to consider the effect of large density gradient on noise generation. All these RANS-based jet noise prediction models are based on the k−ε model and can give relatively accurate predictions of the jet radiative noise at the sideline, where the fine-scale turbulent noise is dominant. In 2011, Leib & Goldstein [11] proposed a hybrid jet noise model, which is also based on the k−ε model. But, the quasi-normal and axisymmetric assumptions are used to account for the anisotropy of jet noise Source. With a set of empirical constants, the predictions of Leib & Goldstein's hybrid jet noise model [12] were in reasonably good agreement with experiment data at observation angles of 90° and downstream 150° but were found to be in error at intermediate angles. More recently, Karabasov *et al*. [13] determined the empirical constants of an RANS-based jet noise prediction method through comparison with correlations obtained from the large eddy simulations of jet noise. For unheated, moderate Mach number jets, Karabasov *et al*.'s model gives encouraging prediction results, even at angles close to the jet axis.

The present work is concerned with the problem of predicting the large-scale turbulent jet noise based on an RANS simulation. To model the anisotropic characteristics of jet noise source, the Reynolds stress is used in place of the turbulent kinetic energy. And a modified LRR-*ω* turbulence model [11,14–16] is used to calculate the Reynolds stress of the jet mean flow. Based on a series of measurements of jet flow turbulence, numerical and experimental jet noise data, the paper establishes an anisotropic turbulence noise model based on the accurate calculation of mean flow field, turbulence information and the Green's function. The relevant empirical coefficients were determined from publicly available experiment data. Finally, comparisons between predicted results and acoustic data were carried out to verify the accuracy of the new anisotropic source model.

## 2. Formulation

The modelling starts from Goldstein's generalized acoustic analogy [13,17], the Navier–Stokes equations are rearranged into Linearized Euler Equations for the propagating quantities, with nonlinear terms representing the analogous acoustic sources. This formulation provides a consistent framework in which the convection effects on acoustic sources and the refraction effects on the sound can be captured. Unlike the sources in previous analogies, the sources in this formulation have zero time average as would be expected from true acoustic sources

2.1 $$\begin{array}{rl} & \frac{\partial \rho^{\prime }}{\partial t}+\frac{\partial }{\partial x_{j}}(\rho^{\prime }{\tilde{U}}_{j}+u_{j}^{\prime })=0\\ & \frac{\partial u_{i}^{\prime }}{\partial t}+\frac{\partial }{\partial x_{j}}({\tilde{U}}_{j}u_{i}^{\prime })+\frac{\partial p^{\prime }}{\partial x_{i}}+u_{j}^{\prime }\frac{\partial {\tilde{U}}_{i}}{\partial x_{j}}-\frac{\rho^{\prime }}{\bar{\rho }}\frac{\partial {\tilde{\tau }}_{ij}}{\partial x_{j}}=\frac{\partial }{\partial x_{j}}T_{ij}^{\prime }\;i=1,\ldots 3\\ & \frac{1}{\left(\gamma -1\right)}\left(\frac{\partial p^{\prime }}{\partial t}+\frac{\partial }{\partial x_{j}}(p^{\prime }{\tilde{U}}_{j})\right)+\frac{\partial }{\partial x_{j}}(u_{j}^{\prime }\tilde{h})+p^{\prime }\frac{\partial {\tilde{U}}_{j}}{\partial x_{j}}-\frac{u_{i}^{\prime }}{\bar{\rho }}\frac{\partial {\tilde{\tau }}_{ij}}{\partial x_{j}}=Q \end{array}\}$$

where $\bar{\left(\;\right)}$ represents a time average, $\tilde{\left(\;\right)}$ a Favre average and single and double primes represent the corresponding variation about the mean, $\bar{p}$ and $\bar{\rho }$ are the time-averaged pressure and density, ${\tilde{U}}_{i}$ and $\tilde{h}$ are the Favre-averaged velocity and enthalpy, respectively, pulsatile variables $p^{\prime },\rho^{\prime },u_{i}^{\prime \prime }\text{ , }h^{\prime \prime }$ and $u_{i}^{\prime }$ are defined as

2.2 $$p^{\prime }=p-\bar{p}\;\rho^{\prime }=\rho -\bar{\rho }\;u_{i}^{\prime \prime }=u_{i}-{\tilde{U}}_{i}\;h^{\prime \prime }=h-\tilde{h}\;u_{i}^{\prime }=\rho u_{i}^{\prime \prime }\text{.}$$

The source terms of generalized acoustic analogy function are

2.3 $$\begin{array}{rl} T_{ij}^{\prime } & =-(\rho u_{i}^{\prime \prime }u_{j}^{\prime \prime }-\bar{\rho u_{i}^{\prime \prime }u_{j}^{\prime \prime }})\\ \;\text{and}\;Q & =-{\tilde{U}}_{i}\frac{\partial T_{ij}^{\prime }}{\partial x_{j}}+\frac{1}{2}\delta_{\text{ij}}\left[\frac{\text{D}T_{ij}^{\prime }}{D\tau }+\frac{\partial {\tilde{U}}_{k}}{\partial x_{k}}T_{ij}^{\prime }\right]-\frac{\partial }{\partial x_{i}}(\rho u_{i}^{\prime \prime }h_{0}^{\prime \prime }-\bar{\rho u_{i}^{\prime \prime }h_{0}^{\prime \prime }}), \end{array}\}$$

where $h_{0}^{\prime \prime }$ is the perturbation of Favre-averaged stagnation enthalpy

2.4 $$h_{0}^{\prime \prime }=h^{\prime \prime }+{\tilde{U}}_{i}u_{i}^{\prime \prime }+\frac{1}{2}u_{ii}^{\prime \prime }.$$

The wave propagation problem is solved in the frequency domain using an adjoint Green function method, the Fourier transform of the adjoint Green function [9,15], satisfying the adjoint equations

2.5 $$\begin{array}{rl} & i\Omega {\hat{G}}_{0}+{\tilde{U}}_{j}\frac{\partial {\hat{G}}_{0}}{\partial x_{j}}+\frac{{\hat{G}}_{i}}{\bar{\rho }}\frac{\partial {\tilde{\tau }}_{ij}}{\partial x_{j}}=0\\ & i\Omega {\hat{G}}_{i}+\frac{\partial {\hat{G}}_{0}}{\partial x_{i}}+{\tilde{U}}_{j}\frac{\partial {\hat{G}}_{i}}{\partial x_{j}}-{\hat{G}}_{j}\frac{\partial {\tilde{U}}_{\text{j}}}{\partial x_{i}}+\tilde{h}\frac{\partial {\hat{G}}_{4}}{\partial x_{i}}+\frac{{\hat{G}}_{4}}{\bar{\rho }}\frac{\partial {\tilde{\tau }}_{ij}}{\partial x_{j}}=0\\ & i\Omega {\hat{G}}_{4}+{\tilde{U}}_{j}\frac{\partial {\hat{G}}_{4}}{\partial x_{j}}+(\gamma -1)\left(-{\hat{G}}_{4}\frac{\partial {\tilde{U}}_{\text{j}}}{\partial x_{j}}+\frac{\partial {\hat{G}}_{i}}{\partial x_{j}}\right)=\delta (x-y). \end{array}\}$$

By means of the adjoint Green's function, the pressure field generated by the source terms on the right side of equation (2.1) can be expressed as

2.6 $$p(y,t)=-\int {∭}_{V_{\infty }(y)}\left({\hat{G}}_{i}(y,t,x,t_{1})\frac{\partial T_{ij}^{\prime }(x,t_{1})}{\partial x_{j}}+{\hat{G}}_{4}(y,t,x,t_{1})Q(x,t_{1})\right){\text{d}}^{3}x\text{d}t_{1}.$$

The space–time Green's function is related to the time harmonic Green's function by the Fourier inverse transform. Then, we have

2.7 $${\hat{G}}_{i}(y,t,x,t_{1})=\int_{-\infty }^{+\infty }{\hat{G}}_{i}(y,x,\Omega )\exp[\left(-i\Omega )(t-t_{1}\right)]\text{d}\Omega ,$$

where Ω is the angular frequency.

Then equation (2.6) can be expressed as

2.8 $$\begin{array}{r} p(y,t)=-\int \int \int \int \int_{V_{\infty }(y)}\left({\hat{G}}_{i}(y,x,\Omega )\frac{\partial {\hat{T}}_{ij}^{\prime }(x,t_{1})}{\partial x_{j}}+{\hat{G}}_{4}(y,x,\Omega )Q(x,t_{1})\right)\exp[\left(-i\Omega )(t-t_{1}\right)]\text{d}\Omega {\text{d}}^{3}x\text{d}t_{1}. \end{array}$$

In the generalized acoustic analogy equation, the source term on the right side of the momentum equation is only related to the turbulent pressure, and the source term on the right side of the energy equation is related not only to the turbulent fluctuations but also to the average flow velocity. For the unheated jets considered in this paper, we will assume that the last term in (2.3) is negligible. And in this paper, the shear stresses are neglected, only Reynolds normal stress-related terms are considered as the noise sources terms. With these approximations, *Q* is reduced to:

2.9 $$\begin{array}{rl} & T_{ij}^{\prime }(x,\tau )\to -(\rho u_{i}^{\prime \prime }u_{j}^{\prime \prime }-\bar{\rho }\tilde{u_{i}^{\prime \prime }u_{j}^{\prime \prime }})\delta_{ij}\\ & Q(x,\tau )\to -{\tilde{U}}_{i}\frac{\partial T_{ij}^{\prime }}{\partial x_{j}}\delta_{ij}+\frac{1}{2}\delta_{ij}\left[\frac{\text{D}T_{ij}^{\prime }}{D\tau }+\frac{\partial {\tilde{U}}_{k}}{\partial x_{k}}T_{ij}^{\prime }\right]. \end{array}\}$$

For the jet flow problem, it is assumed that the jet flow is a nearly parallel flow in the model: $\partial \tilde{U}\text{ /}\partial x=0$, $\tilde{V}=0,$ $\tilde{W}=0$. Then, by integration by parts, the source term *Q* is reduced to

2.10 $$Q(x,\tau )\to -\frac{\partial \tilde{U}T_{11}^{\prime }(x,\tau )}{\partial x}+\frac{1}{2}\frac{\partial T_{ii}^{\prime }(x,\tau )}{\partial \tau }.$$

By substituting equation (2.10) into equation (2.8)

2.11 $$\begin{array}{rl} p(y,\text{t}) & =-\int \int \int_{V_{\infty }(y)}\left({\hat{G}}_{i}(y,x,\Omega )\frac{\partial T_{ij}^{\prime }(x,t_{1})}{\partial x_{j}}\delta_{ij}+{\hat{G}}_{4}(y,x,\Omega )\left[\frac{-i\Omega }{2}T_{ii}^{\prime }(x,t_{1})-\frac{\partial \tilde{U}T_{11}^{\prime }(x,t_{1})}{\partial x}\right]\right)\\ & \;\times \exp[\left(-i\Omega )(t-t_{1}\right)]\text{d}\Omega {\text{d}}^{3}x\text{d}t_{1}. \end{array}$$

Based on Gauss' Law, equation (2.13) can be written as

2.12 $$\begin{array}{rl} p(y,t) & =\int \int \int_{V_{\infty }(y)}\left(\left(\frac{\partial {\hat{G}}_{i}(y,x,\Omega )}{\partial x_{j}}-\frac{i\Omega }{2}{\hat{G}}_{4}(y,x,\Omega )\right)T_{ij}^{\prime }(x,t_{1})\delta_{ij}+\frac{\partial {\hat{G}}_{4}(y,x,\Omega )}{\partial x_{1}}\tilde{U}T_{11}^{\prime }(x,t_{1})\right)\\ & \;\times \exp[\left(-i\Omega )(t-t_{1}\right)]\text{d}\Omega {\text{d}}^{3}x\text{d}t_{1} \end{array}$$

In this paper, it is assumed that the sound sources are independent. By means of equation (2.12), the autocorrelation function for a point in the acoustic far-field can be formed as

2.13 $$\begin{array}{rl} \left\langle p(y,t)p(y,t+\tau )\right\rangle & =\int_{-\infty }^{\infty }\cdots \int_{-\infty }^{\infty }\left\{\frac{\partial {\hat{G}}_{4}(y,x_{1},\Omega_{1})}{\partial x}\frac{\partial {\hat{G}}_{4}(y,x_{2},\Omega_{2})}{\partial x}\langle \tilde{U}T_{11}^{\prime }(x_{1},t_{1})\cdot \tilde{U}T_{11}^{\prime }(x_{2},t_{2})\right\rangle \\ & \;+\left(\frac{\partial {\hat{G}}_{i}(y,x_{1},\Omega_{1})}{\partial x_{j}}-\frac{i\Omega_{1}}{2}{\hat{G}}_{4}(y,x_{1},\Omega_{1})\right)\\ & \;\times \left(\frac{\partial {\hat{G}}_{i}(y,x_{2},\Omega_{2})}{\partial x_{j}}-\frac{\text{i}\Omega_{2}}{2}{\hat{G}}_{4}(y,x_{2},\Omega_{2})\right)\langle T_{ij}^{\prime }(x_{1},t_{1})T_{ij}^{\prime }(x_{2},t_{2})\rangle \delta_{ij}\}\\ & \;\times \;\exp[-i\Omega_{1}(t-t_{1})-i\Omega_{2}(t-t_{2})-i\Omega_{2}\tau ]{\text{d}}^{3}x_{1}{\text{d}}^{3}x_{2}\text{d}\Omega_{1}\text{d}\Omega_{2}\text{d}t_{1}\text{d}t_{1} \end{array}$$

In equation (2.13), <> is the ensemble average.

The spectral density of the radiated sound S(y,Ω) is the Fourier transform of the autocorrelation function

2.14 $$\text{S}(y,\Omega )=\int_{-\infty }^{-\infty }\langle p(y,t)p(y,t+\tau )\rangle {\text{e}}^{i\Omega \tau }\;\text{d}\tau ,$$

By using

2.15 $$\int_{-\infty }^{-\infty }\exp(i(\Omega -\Omega_{2})\tau )\text{d}\tau =2\pi \delta (\Omega -\Omega_{2}).$$

It is straightforward to derive the following formula for S(y,Ω) from equation (2.14):

2.16 $$\begin{array}{rl} S(y,\Omega ) & =\int_{-\infty }^{\infty }\cdots \int_{-\infty }^{\infty }\{\frac{\partial {\hat{G}}_{4}(y,x_{1},\Omega_{1})}{\partial x}\frac{\partial {\hat{G}}_{4}(y,x_{2},\Omega_{2})}{\partial x}\langle \tilde{U}T_{11}^{\prime }(x_{1},t_{1})\tilde{U}T_{11}^{\prime }(x_{2},t_{2})\rangle \\ & \;+\;\left(\frac{\partial {\hat{G}}_{i}(y,x_{1},\Omega_{1})}{\partial x_{j}}-\frac{\text{i}\Omega_{1}}{2}{\hat{G}}_{4}(y,x_{1},\Omega_{1})\right)\\ & \;\times \left(\frac{\partial {\hat{G}}_{i}(y,x_{2},\Omega_{2})}{\partial x_{j}}-\frac{\text{i}\Omega_{2}}{2}{\hat{G}}_{4}(y,x_{2},\Omega_{2})\right)\langle T_{ij}^{\prime }(x_{1},t_{1})T_{ij}^{\prime }(x_{2},t_{2})\rangle \delta_{ij}\}\\ & \;\times \exp[-i(\Omega_{1}+\Omega_{2})t+-i\Omega_{1}t_{1}+i\Omega_{2}t_{2}](\Omega -\Omega_{2}){\text{d}}^{3}x_{1}{\text{d}}^{3}x_{2}\text{d}\Omega_{1}\text{d}\Omega_{2}\text{d}t_{1}\text{d}t_{2} \end{array}$$

For the far-field sound power formula (equation (2.16)), the calculation of adjoint Green's function and the modelling of $\left\langle T_{ij}^{\prime }(y,t)\cdot T_{ij}^{\prime }(y,t+\tau )\right\rangle$ and $\left\langle \tilde{U}T_{11}^{\prime }(x_{1},t_{1})\cdot \tilde{U}T_{11}^{\prime }(x_{2},t_{2})\right\rangle$ are the key problems for RANS-based jet noise prediction method. In this paper, to account for the refraction effects of jet mean flow, the adjoint Green's functions (equation (2.5)) are solved computationally. And the adjoint Green's function problem can be recast into a wave scattering. The sum of the scattered waves and the incident wave satisfies the homogeneous form of equation (2.5). One advantage of solving the wave-scattering problem instead of the original problem is that there is no source singularity (the delta function at the source) to deal with. This is especially helpful when a computational aeroacoustics method is used.

Let

2.17 $${\hat{G}}_{i}={\hat{G}}_{i}^{\prime }+{\hat{G}}_{i,\text{in}}\;(i=0,1,2,3,4).$$

By substituting equation (2.17) into equation (2.5), the equation is written in the operator form:

2.18 $$i\Omega ({\hat{G}}_{i}^{\prime }+{\hat{G}}_{i,\text{in}})+L({\hat{G}}_{i}^{\prime }+{\hat{G}}_{i,\text{in}})=0.$$

By placing the incident wave term on the right side of the equation, we obtain:

2.19 $$i\Omega {\hat{G}}_{i}+L({\hat{G}}_{i}^{\prime })=-i\Omega {\hat{G}}_{i,\text{in}}-L({\hat{G}}_{i,\text{in}}).$$

Equation (2.19) is discretized according to the seven-point stencil Dispersion-Relation-Preserving (DRP [18]) finite difference scheme (Tam & Webb) in a rectangular computation domain. The outer boundary of the computation domain is taken to be where the mean flow velocity $\tilde{U}(x,y,z)$ differs from far-field velocity ${\tilde{U}}_{\infty }$ by less than 0.1% of axis velocity ${\tilde{U}}_{j}$. And the computation domain is surrounded by the perfect matched layer (PML) boundary condition [19]. The PML has a thickness of 20 mesh points. In this layer, the flow variables are split into two parts (the split variable method). One part of the variables will be assigned the values of the incident wave. The other part is the scattered waves. The scattered waves are absorbed by the PML boundary condition. [19].

With calculated adjoint Green's functions, the accuracy of prediction method is directly related to the source modelling function. In formula (2.16), the sound source is mainly divided into two kinds of source items. The first item is related to turbulent fluctuations, which can be considered as fine-scale turbulent structure noise source. And the second source term contains the effect of the average flow which can be considered to be the large-scale turbulent noise source.

2.20 $$\begin{array}{rl} R_{ij}(x_{1},x_{2},t_{1},t_{2}) & =\langle T_{ij}^{\prime }(x_{1},t_{1})T_{ij}^{\prime }(x_{2},t_{2})\rangle \\ \;\text{and}\;Q(x_{1},x_{2},t_{1},t_{2}) & =\langle \tilde{U}(x_{1})T_{11}^{\prime }(x_{1},t_{1})\cdot \tilde{U}(x_{2})T_{11}^{\prime }(x_{2},t_{2})\rangle . \end{array}\}$$

Therefore, how to establish an accurate and reliable sound source model is the focus of this paper.

## 3. Anisotropic fine-scale source modelling

For the fine-scale turbulent noise source which contains only the pulsation term, the sound source modelling function refers to the Gaussian function of the TA method, and on the basis of considering the influence of the anisotropic Reynolds stress on the sound source, the sound source modelling is established. The modelling function is given as follows:

3.1 $$R_{ij}=\langle T_{ij}^{\prime }(x_{1},t_{1})T_{ij}^{\prime }(x_{2},t_{2})\rangle =A_{\text{fine}}{\left(\rho \bar{u_{i}^{\prime }u_{j}^{\prime }}\right)}^{2}\exp\left\{-\frac{|\xi |}{\bar{u}\tau_{\text{s}}}-\ln2\left[\frac{{\left(\xi -U\tau \right)}^{2}}{l_{\text{s}x}^{2}}+\frac{\eta^{2}}{l_{\text{s}y}^{2}}+\frac{\zeta^{2}}{l_{\text{s}z}^{2}}\right]\right\},$$

where *ξ* = *x*_1_ − *x*_2_, *η* = *y*_1_ − *y*_2_, *ζ* = *z*_1_ − *z*_2_, *τ* = *t*_1_ − *t*_2_, $\tau_{\text{s}}$ is the time scale of fine-scale turbulence, and $l_{\text{s}}$ is the turbulent length scale.

In the previous prediction model (TA [9], JeNo [20]), the time scale $\tau_{\text{s}}$ and the length scale $l_{\text{s}}$ were obtained through the *k*–*ϵ* turbulence model. In the present work, to model the anisotropic characteristics of the jet noise source, the LRR-*ω* Reynolds stress model is used to calculate the jet mean flow. It is generally known that the standard model does poorly in predicting the mean flow of jets. Xu *et al.* [21] recognized the problem of applying the standard model to jet flow simulation. They proposed modifying the LRR-*ω* model coefficients and showed convincingly, by a comparison with a large set of jet flow data over the jet Mach number range of 0.4–1.0 and temperature ratio range of 1.0 (cold jet)–2.7, that their modified model is reliable and accurate. In this work, the modified LRR-*ω* model of [11] is used. The model provides not only turbulence kinetic energy *k*, but also Reynolds stresses, which are very important to anisotropic source modelling. And specific dissipation rate *ω* is provided instead of dissipation rate *ϵ*.

With Reynolds stress and specific dissipation rate ω known, it is possible to form length scales $l_{\text{s}}$ and a decay time $\tau_{\text{s}}$ of the turbulence as follows:

3.2 $$\tau_{\text{s}}=c_{\tau }\left(\frac{1}{\omega }\right),$$

and

3.3 $$\begin{array}{rl} l_{\text{s}x} & =c_{l-\text{fine}}\left(\frac{{\bar{u^{\prime }u^{\prime }}}^{1/2}}{\omega }\right)\\ l_{\text{s}y} & =c_{l-\text{fine}}\left(\frac{{\bar{v^{\prime }v^{\prime }}}^{1/2}}{\omega }\right)\\ \;\text{and}\;l_{\text{s}y} & =c_{l-\text{fine}}\left(\frac{{\bar{w^{\prime }w^{\prime }}}^{1/2}}{\omega }\right), \end{array}\}$$

where $\bar{u^{\prime }u^{\prime }},\bar{v^{\prime }v^{\prime }},\bar{w^{\prime }w^{\prime }}$ are the Reynolds normal stresses, ω is the specific dissipation rate of the LRR-*ω* turbulent model, $c_{\tau }$ and $c_{l-\text{fine}}$ are the empirical constants of time scale and length scale, respectively.

In the modelling, it is assumed that the spatial-temporal correlation of the jet noise source is consistent with the spatial-temporal correlation of the jet turbulence itself. Therefore, the empirical coefficients of time and space length scales are obtained by fitting the spatial-temporal correlation experimental turbulence data. In the present work, the empirical coefficients are

$$A_{\text{fine}}=6.56,C_{l-\text{fine}}=1.8,C_{\tau }=1.69.$$

**Figure 1.**
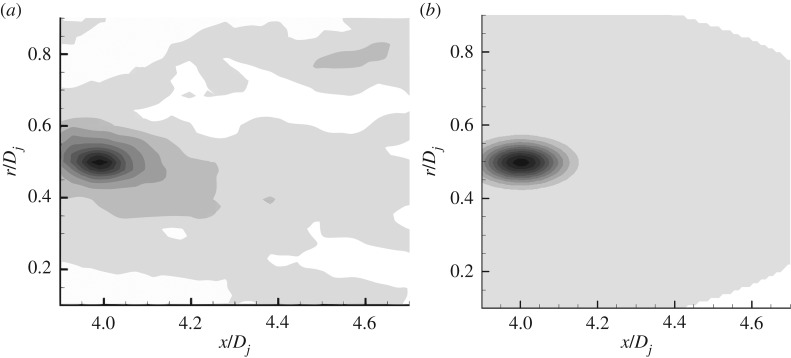
Spatial correlation contour of Reynolds stress at decay time *τ* = 0. (*a*) Pokora & McGuirk's data [22]. (*b*) Anisotropic source model.

**Figure 2.**
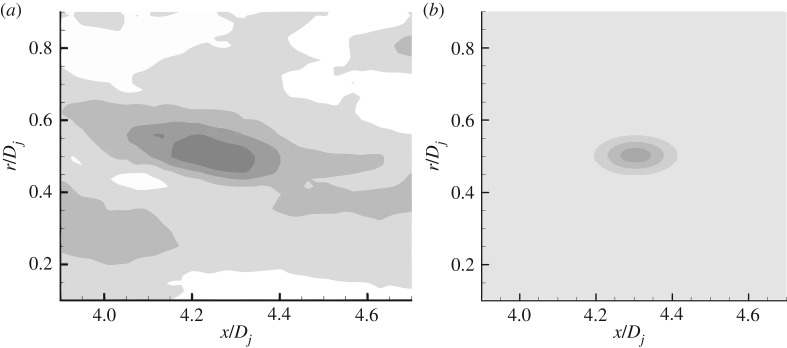
Spatial correlation contour of Reynolds stress at decay time *τ* = 0.6*D_j_*/*U*_jet_. (*a*) Pokora & McGuirk's data [22]. (*b*) Anisotropic source model.

In this paper, a series of comparisons with experimental data are carried out on the established turbulent noise source model and related empirical coefficients. Figure 1 shows the comparison of the Reynolds stress spatial correlation contour in the jet shear layer. For the jet exit acoustic Mach number of 0.8, at the point (*r* = 0.5D, *x* = 4D), the modelled time-spatial correlation of axial Reynolds stress is compared with the experiment data of Pokora & McGuirk [22]. The comparison shows that the source model established in this paper can exhibit the anisotropy property of jet shear layer. The correlation in the axial direction is significantly stronger than the radial correlation, which is consistent with the experimental results of Pokora & McGuirk [22]. Figure 2 shows the comparison of the Reynolds stress spatial correlation contour at decay time *τ* = 0.6*D_j_*/*U*_jet_. The results show that due to the convection effect of the average flow of the jet, the amplitude position of the spatial correlation function moves downstream of the jet, and the magnitude of the correlation gets smaller, while still exhibiting anisotropic characteristics. The turbulent noise source model of the jet shear layer developed in this paper is roughly the same in terms of spatio-temporal correlation characteristics as the experimental results.

**Figure 3.**
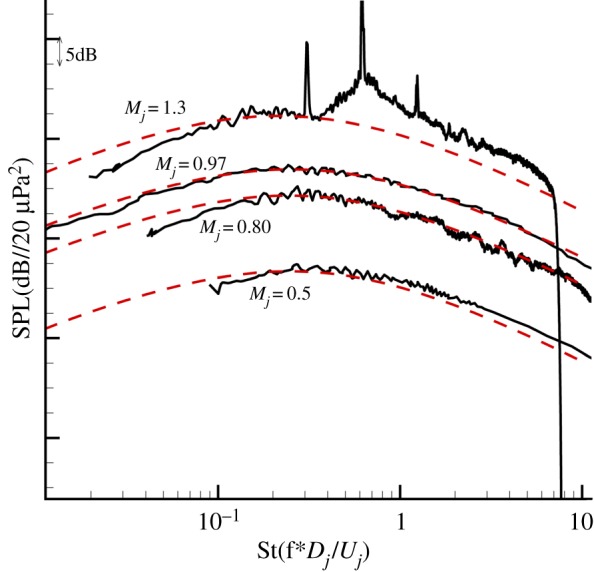
Comparison of spectra at 90° to the jet axis and *R* = 100*D.* (Online version in colour.)

Based on the anisotropic model proposed above, combined with the calculated results of the jet mean flow and adjoint Green's functions, the developed models are applied to predict the noise from cold jets with different jet exit acoustic Mach numbers (Ma = 0.5–1.3). Figure 3 shows the comparison between the predicted results and experiment data of the observer located at 100 diameter and 90° to nozzle exit. The comparison confirmed that the predicted far-field noise spectra have an excellent agreement with experiment data, except for the incompletely expanded jet with Mach number 1.3. Because of the broadband shock-associated noise, the experiment data are about 5 dB larger than the prediction results in high frequency.

**Figure 4.**
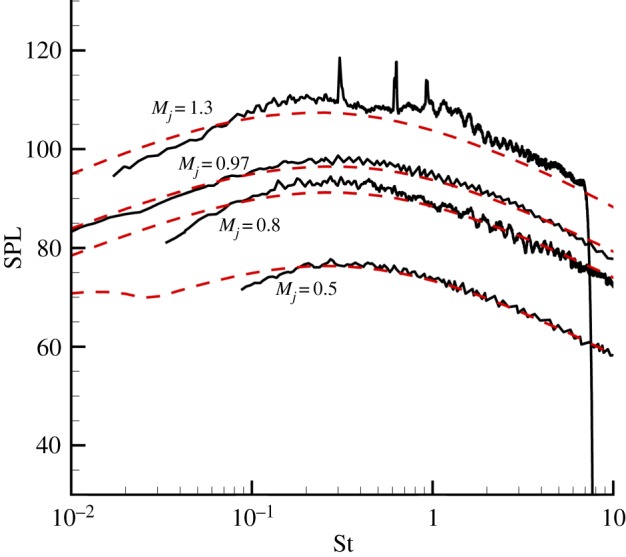
Comparison of spectra at 60° to the jet axis and *R* = 100*D.* (Online version in colour.)

Figure 4 shows the comparison between the predicted results and experimental data of the observer located at 100 diameter and 60° to nozzle exit. The fine-scale turbulent noise is still dominant in that direction. Therefore, only the fine-scale turbulent noise model is used to predict the noise for the observer at 60°. The comparison indicates that the predicted far-field noise spectra still have a good agreement with experiment data. And for incompletely expanding jet, with jet exit acoustic Mach numbers of 1.3, the broadband shock-associated noise decreased when the observer moves to the downstream. The prediction results of the observer at 60° fit the experiment data better than at 90°. The experiment data are about 3 dB larger than the prediction results in high-frequency band, dominated by the broadband shock-associated noise.

## 4. Large-scale turbulent noise prediction

For large-scale turbulent noise source terms, coherent with the average flow and turbulent pulsation, similar noise source terms can also be found in the MGBK and JeNo methods based on the Lilley equation, considered as shear noise sources. While both the MGBK and JeNo method are only modulated as the pulsating quadrupole source, and the average velocity in the source term is considered to affect only the acoustic propagation as part of the Green's function solution.

However, the study considers that the large-scale turbulent noise source has a great relationship with the average flow, which is mainly reflected in the fact that the length scale of the large-scale turbulent should be larger than the fine-scale turbulence length scale, and the directivity of the large-scale structure radiation noise is more than that of the fine-scale turbulence noise. It is obvious. Therefore, the following large-scale turbulence noise source modelling function is established

4.1 $$\begin{array}{rl} \text{Q}(\xi ) & =\langle \hat{UT_{11}^{\prime }}({\bar{x}}_{1},t)\cdot \hat{UT_{11}^{\prime }}({\bar{x}}_{2},t+\tau )\rangle \\ & =A_{\text{large}}{\left(\rho \text{U}\bar{u^{\prime }u^{\prime }}\right)}^{2}\exp\left\{-\frac{|\xi |}{\bar{u}\tau_{\text{s}}}-\frac{\ln2}{l_{s-\mathrm{large}}^{2}}[{\left(\xi -\bar{u}\tau \right)}^{2}+\eta^{2}+\zeta^{2}]\right\} \end{array}$$

where $A_{\text{large}}$ is the empirical constant of large-scale noise source intensity, and $l_{s}$ is the length scale of modelled large-scale turbulence noise source.

The large-scale turbulent noise source is the coherent structure of average flow and turbulence. The length scale of the average flow convection is much larger than that of the fine-scale turbulence structure and is related to the average flow velocity. Therefore, the length scale of large-scale turbulent noise sources in this paper is defined as:

4.2 $$l_{\text{large}}=c_{l-\text{large}}\frac{\tilde{u}}{c_{\infty }}\left(\frac{{\bar{u^{\prime }u^{\prime }}}^{1/2}}{\omega }\right),$$

where $C_{l-\text{large}}$ is the empirical constant of length scale for large-scale noise source, and ω is the specific dissipation rate of the LRR-*ω* turbulent model. In the present work, by fitting the experimental data, the empirical coefficients $A_{\text{large}}$ and $C_{l-\text{large}}$ are given by: $A_{\text{large}}=49.2,C_{l-\text{large}}=6.12.$

It is assumed that the noise generated by large-scale turbulence propagates only to the axial direction of the jet. And the refraction effects of the jet shear layer cause the deflection of the radiated sound waves away from the jet flow direction. Under this assumption, the far-field acoustic pressure power spectral density of large-scale turbulent noise can be expressed as

**Figure 5.**
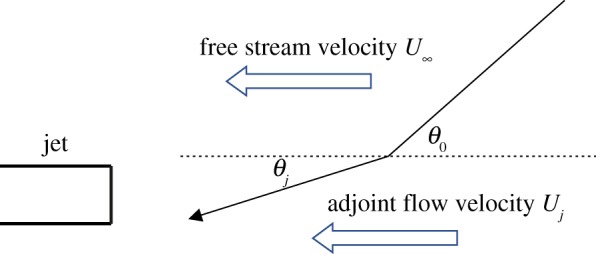
Mean flow refraction. (Online version in colour.)

4.3 Slarge(y,Ω)=∫∫∫V(x)∫∫∫V(ξ){|∂G^4(y,x,Ω)∂x|2e−ikξ⟨UT11′^(x,t)⋅UT11′^(x,t+τ)⟩×δ(θ,y,x,Ω)−0)δ(0)dξdx|∂G^4(y,x,Ω)∂x|2},

where ${\hat{G}}_{4}(y,x,\Omega )$ is the adjoint pressure-like quantity of adjoint Green's function, Ω is the angular frequency and θ(y,x,Ω) is the radiation angle of adjoint acoustic wave. In consideration of the assumption that the noise generated by large-scale turbulence propagates only to the axial direction of the jet, the source model of large structure turbulent noise will be activated only where the radiation angle θ(y,x,Ω) equals 0. As shown in figure 5, the radiation angle changes, when the adjoint wave propagates through the adjoint jet mean flow.

In the present work, the adjoint wave radiation angle inside the jet flow is calculated in a simple way. The axial wavelength outside the jet flow is

4.4 $$\lambda_{x}=\frac{1}{f}\left\{\frac{c_{\infty }}{\text{cos}\theta_{\infty }}+U_{\infty }\right\}.$$

The axial wavelength inside the jet flow is

4.5 $$\lambda_{x}=\frac{1}{f}\left\{\frac{c_{j}}{\text{cos}\theta_{j}}+U_{j}\right\},$$

where $c_{\infty }$ and *c_j_* are the ambient acoustic velocity and acoustic velocity inside the jet flow. $U_{\infty }$ and *U_j_* are the free stream velocity and adjoint flow velocity, respectively, inside the jet flow.

The axial wavelength must be equal at the interface; therefore,

4.6 $$\lambda_{x}=\frac{1}{f}\left\{\frac{c_{\infty }}{\text{cos}\theta_{\infty }}+U_{\infty }\right\}=\frac{1}{f}\left\{\frac{c_{j}}{\text{cos}\theta_{j}}+U_{j}\right\}.$$

Then

4.7 $$\theta_{j}=\text{co}{\text{s}}^{-1}\left\{\frac{c_{j}}{c_{\infty }\text{/cos}\theta_{\infty }+U_{\infty }-U_{j}}\right\}.$$

**Figure 6.**
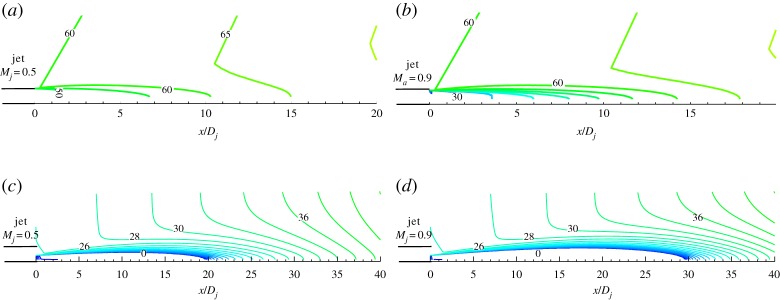
Mean flow refraction effects. (Online version in colour.)

The flight effect is not considered in this paper. In the following text, free stream velocity *U*_∞_ = 0. Figure 6 shows the contour of radiation angle for different jet Mach numbers and different observer locations (adjoint source location). As shown in figure 6*a,b* for both Mach number 0.5 and 0.9 cold jet, the observer is located at 100*D_j_* and 60° to the jet axis. The radiation angle of the adjoint wave is always greater than zero, which means that the noise generated by large-scale turbulence will not propagate to the observer point located at 60° to the jet axis and 100 diameters to the jet exit.

As shown in figure 6*c,d* for both Mach number 0.5 and 0.9 cold jet, the observer is located at 100*D_j_* and 60° to the jet axis. The radiation angle of adjoint wave decreased to 0, when the adjoint wave propagates through the jet shear layer, which means that the observer, located at 100*D_j_* and 60° to the jet axis, will be suffered by large-scale turbulent noise.

**Figure 7.**
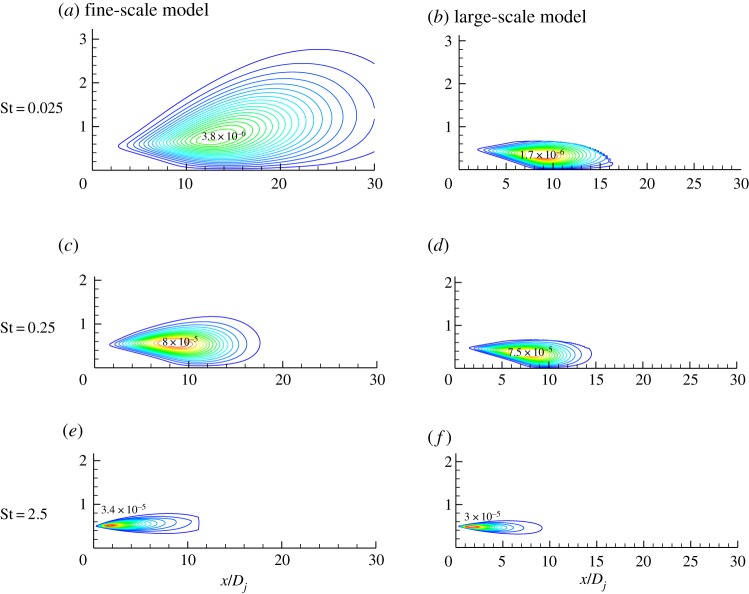
Contours of contributions to acoustic pressure power spectral density (*M_j_* = 0.5). (*a*,*c*,*e*) Fine-scale model. (*b*,*d*,*f*) Large-scale model. (Online version in colour.)

**Figure 8.**
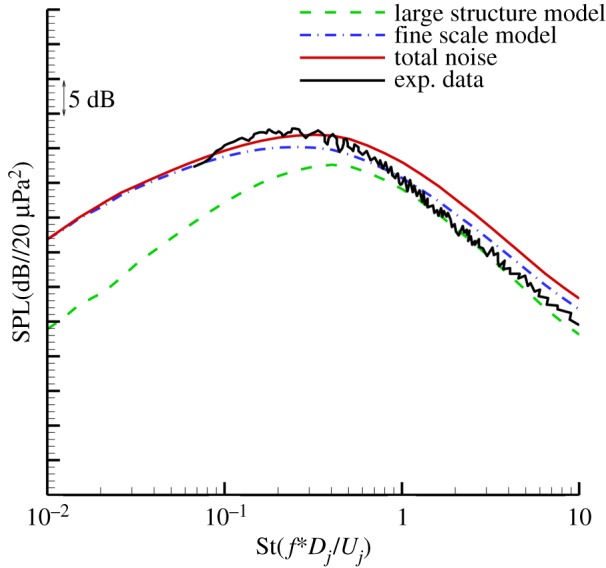
Comparison of spectra at 30° to the jet axis and *R* = 100*D* (Ma = 0.5). (Online version in colour.)

Figure 7 shows the predicted results of a large- and fine-scale noise source with a Mach number of 0.5 at different frequencies. The sound source modelling results show that for the Mach number 0.5 jet in the downstream 30° direction, the intensity of the fine-scale turbulent noise source is still greater than the large-scale turbulent noise intensity, and the fine-scale turbulent noise sources occupy a dominant position at each frequency. Figure 8 shows the comparison of the predicted results of the far-field noise spectrum in the 30° downstream direction with the experimental results. Figure 8 includes the predicted spectrum of fine-scale turbulent noise and the large-scale turbulent noise prediction spectrum. The final far-field noise prediction spectrum is the result of the combined effect of fine-scale noise and large-scale turbulent noise. The prediction results show that for the jet with the exit Mach number of 0.5, the downstream direction of the jet noise is still dominated by fine-scale turbulent noise. The final prediction results are in good agreement with the experimental results. The prediction results in the high-frequency band are slightly higher than the experimental data, and the overall error is about 3 dB.

**Figure 9.**
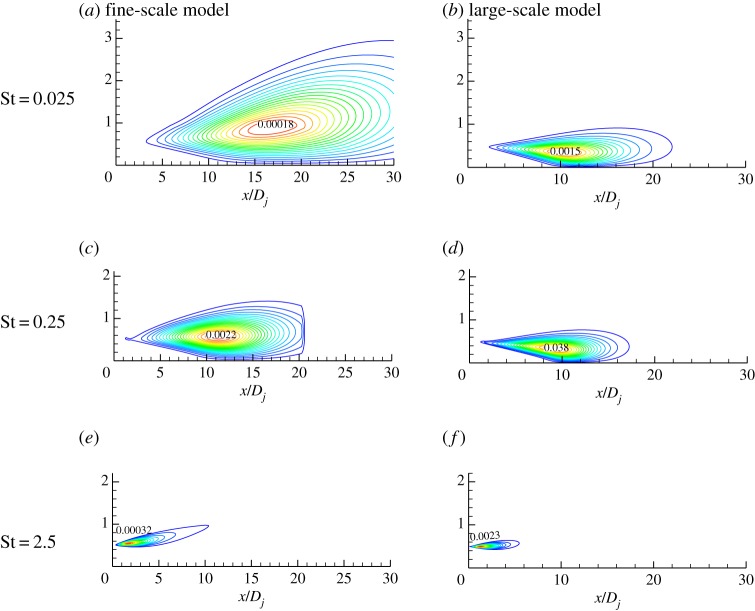
Contours of contributions to acoustic pressure power spectral density (*M_j_* = 0.97). (*a*,*c*,*e*) Fine-scale model. (*b*,*d*,*f*) Large-scale model. (Online version in colour.)

**Figure 10.**
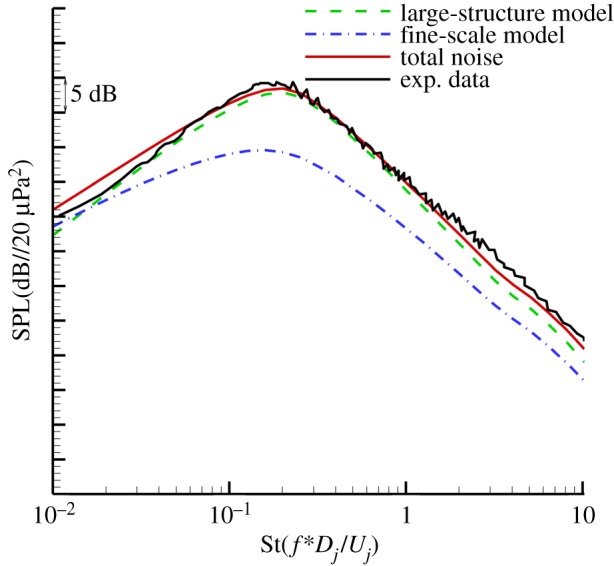
Comparison of spectra at 30° to the jet axis and *R* = 100*D* (*M_j_* = 0.97). (Online version in colour.)

Figure 9 shows the predicted results of a large- and fine-scale noise source from a jet with Mach number 0.97. The sound source modelling results show that for the Mach number 0.97 jet in the downstream 30° direction, the intensity of the large-scale turbulent noise source is greater than the fine-scale turbulent noise intensity, and the large-scale turbulent noise sources occupy a dominant position at each frequency. Figure 10 shows the comparison between the predicted results and experiment data for the observer located at 100 diameter and 30° downstream to the jet exit. Figure 10 includes the predicted spectrum of fine-scale turbulent noise and the large-scale turbulent noise prediction spectrum. The final far-field noise prediction spectrum is the result of the combined effect of fine-scale noise and large-scale turbulent noise. The prediction results show that for the jet with Mach number of 0.97, the downstream direction of the jet noise is dominated by large-scale turbulent noise. The final prediction results are in good agreement with the experimental results.

**Figure 11.**
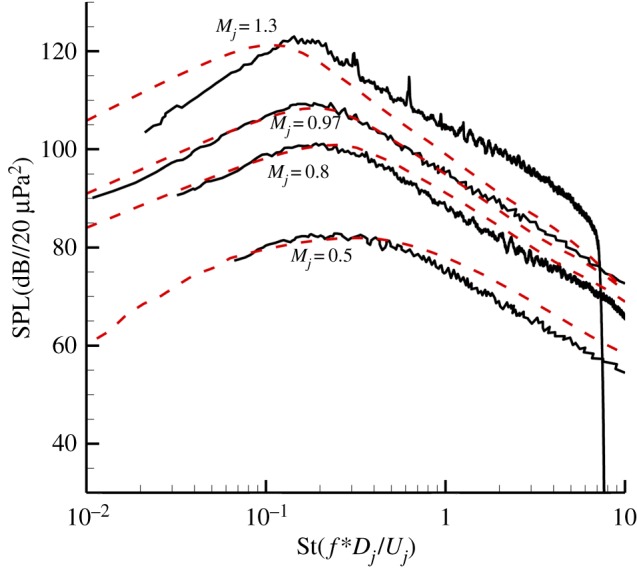
Comparison of spectra at 30° to the jet axis and *R* = 100*D*. (Online version in colour.)

Figure 11 shows the comparison between prediction results and experiment data for jets with different jet exit acoustic Mach numbers (*M_j_* = 0.5–1.3). As discussed before, for a jet with Mach number 0.5, fine-scale turbulent noise is still dominant even for 30° downstream direction. The established anisotropic fine-scale source model can provide accurate results. As the jet velocity increases, the noise from the large structures gradually becomes dominant. For jets with jet exit acoustic Mach numbers of 0.8 and 0.97, the comparisons between prediction results and experiment data show that the large-scale turbulent noise model established in this work also can predict relatively well. While for an incompletely expanding jet with Mach number 1.3, the RANS-based prediction method overpredicts the noise in low frequency and underpredicts in high frequency. These discrepancies could be caused by the source model itself or the calculation of Green's function, which needs further investigation.

## 5. Conclusion

Based on Goldstein's generalized acoustic analogy, the anisotropic fine-scale and large-scale turbulence noise models have been established. To model the anisotropic characteristics of jet noise source, the Reynolds stress is used to replace the turbulent kinetic energy.

The present model is semi-empirical. Both the fine-scale and the large-scale turbulent noise models contain a fair amount of empirical coefficients, which are determined by experimental data. The developed models are applied to predict the noise from cold jets with different jet exit acoustic Mach numbers of 0.5, 0.8, 0.97 and 1.3. The predicted results are compared thoroughly with the experimental data. For the observer located at 90 and 60°, where fine-scale turbulent noise is dominant, the anisotropic model can provide accurate far-field noise spectra. The observer moves to the downstream 30°, suffered by large-scale turbulent noise. For a low-speed jet with Mach number 0.5, which is still dominated by the fine-scale turbulent noise, the prediction results agree with the experiment data well. As the jet exit velocity increases, the noise from the large structures gradually becomes dominant. The comparisons show that the large-scale turbulent noise model established in this work can predict relatively well. For an incompletely expanding jet with Mach number 1.3, the RANS-based prediction method overpredicts the noise in low frequency and underpredicts in high frequency. Further research on both source modelling and calculation of Green's function should be conducted for better jet noise prediction.

## Data accessibility

This article does not contain any additional data.
